# An adenylyl cyclase like-9 gene (*NlAC9*) influences growth and fecundity in the brown planthopper, *Nilaparvata lugens* (Stål) (Hemiptera: Delphacidae)

**DOI:** 10.1371/journal.pone.0189214

**Published:** 2017-12-13

**Authors:** LinQuan Ge, HaoTian Gu, Bo Huang, Qisheng Song, David Stanley, Fang Liu, Guo-Qing Yang, Jin-Cai Wu

**Affiliations:** 1 School of Horticulture and Plant Protection, Yangzhou University, Yangzhou P.R. China; 2 Division of Plant Sciences, University of Missouri, Columbia, MO, United States of America; 3 USDA/Agricultural Research Service, Biological Control of Insects Research Laboratory, Columbia, Missouri, United States of America; Chinese Academy of Agricultural Sciences Institute of Plant Protection, CHINA

## Abstract

The cAMP/PKA intracellular signaling pathway is launched by adenylyl cyclase (AC) conversion of adenosine triphosphate (ATP) to 3', 5'-cyclic AMP (cAMP) and cAMP-dependent activation of PKA. Although this pathway is very well known in insect physiology, there is little to no information on it in some very small pest insects, such as the brown planthopper (BPH), *Nilaparvata lugens* Stål. BPH is a destructive pest responsible for tremendous crop losses in rice cropping systems. We are investigating the potentials of novel pest management technologies from RNA interference perspective. Based on analysis of transcriptomic data, the BPH AC like-9 gene (*NlAC9)* was up-regulated in post-mating females, which led us to pose the hypothesis that *NlAC9* is a target gene that would lead to reduced BPH fitness and populations. Targeting *NlAC9* led to substantially decreased soluble ovarian protein content, yeast-like symbiont abundance, and vitellogenin gene expression, accompanied with stunted ovarian development and body size. Eggs laid were decreased and oviposition period shortened. Taken together, our findings indicated that *NlAC9* exerted pronounced effects on female fecundity, growth and longevity, which strongly supports our hypothesis.

## Introduction

Many extracellular signals, such as hormones, peptides, biogenic amines and prostaglandins, are transduced into intracellular second messengers via G-protein coupled receptors (GPCRs). As the receptors bind to their specific ligands they undergo a conformational change that leads to activation of their coupled G proteins, the heterotrimeric guanine nucleotide-binding proteins. The G proteins activate any of several downstream effectors, one of which is adenylyl cyclase (AC) [1]. Activated AC converts ATP into cyclic adenosine 3',5'-monophosphate (cAMP), which stimulates cAMP-dependent protein kinase (PKA). PKA is one of the major kinases acting in reversible protein phosphorylation mechanisms in which protein functions are regulated by adding one or more phosphate groups to proteins (by kinases) and by removing them (by phosphatases) [2]. The very broad significance of the PKA pathway is indicated by a list of over 100 physiological PKA substrates [3].

The number of PKA substrates draws attention to the significance of AC, which operates in most cells, including insect cells, first discovered over 30 years ago [4]. They have been documented in a wide range of insect tissues, including prothoracic glands of the tobacco hornworm, *Manduca sexta* [5] and silkworm, *Bombyx mori* [6] and brains of *Drosophila melanogaster* and honey bees, *Apis mellifera* [7]. ACs act in a very wide array of cellular functions. For a single example, AC/cAMP/PKA signaling acts in *Drosophila* behavioral changes during alcohol intoxication [8]. ACs also act in several aspects of reproduction, including spermatogenesis in *Drosophila* [8]. Because of their many, central signaling functions in reproduction and other aspects of insect biology, we raised the question of whether silencing AC would influence reproductive biology of insect pests.

The brown planthopper (BPH), *Nilaparvata lugens* Stål (Hemiptera: Delphacidae), is a major insect pest throughout Asian rice growing regions. It is responsible for very large reduction in rice yields (up to 60%), directly by feeding on plant fluids and indirectly as vectors of rice pathogenic viruses [9]. BPH damage is difficult to detect because significant yield loss occurs before plant damage can be registered [10]. The potentials for wide-spread crop losses are exacerbated by two patterns. First, pest resistance and resurgence due to long-term overuse of chemical insecticides and other agricultural chemicals is increasing. Second, BHP has evolved unexpected responses to agricultural chemicals: increased fecundity and populations [11, 12]. These patterns amount to a case-study of the long called for development of novel insect pest management technologies [13].

One novel technology is the concept of regulating pest populations by silencing genes operating at crucial points in insect biology [14], which we have been developing for BHP in which gene-silencing constructs are ingested via the food stream [15–17]. Based on analysis of transcriptomic data, the BPH AC like-9 gene (*NlAC9)* was up-regulated in post-mating females (S1 Table). A central issue in this new technology is identification and selection of the most appropriate target genes. Because AC operates in a very wide range of cell signaling processes, including those directly involved in reproduction, we posed the hypothesis that AC9 is a target gene that would lead to reduced BPH fitness and populations. Here we report on the outcomes of experiments designed to test our hypothesis.

## Materials and methods

### Rice variety and culture

Seeds of susceptible rice (*Oryza sativa* ssp. japonica) cv. Yangjing 805 were sown in cement tanks (height 60 cm, width 100 cm, and length 200 cm) containing standard rice-growing soil at the Yangzhou University Experimental Farm under natural outdoor conditions. Seedlings bearing six leaves were transplanted into 16 cm diameter plastic pots with four hills per pot, three plants per hill. All rice plants were grown to the tillering stage under natural condition for experiments

### Insect culture

BPH used in the experiments were obtained from a stock population at the China National Rice Research Institute (Hangzhou, China), and maintained in a greenhouse under a 16L: 8D photoperiod at 26±2°C, with 70–80% humidity in the ecological lab of Yangzhou University without insecticide application. Prior to the experiments, the insect colony was allowed to reproduce two generations in cement tanks (60×100×200 cm) under natural conditions.

### dsRNA

We created *NlAC9*-specific primers and amplified a 579-bp (1437-2015bp) *NlAC9* cDNA fragment (NLU001904) according to *NlAC9* sequence (S2 Table and S1 File) using forward and reverse primers containing the T_7_ primer sequence at the 5′-ends (Table 1). The amplification program was 35 cycles of 95°C for 40 s, 56°C for 40 s and 72°C for 1 min, with a final extension step at 72°C for 10 min. The sequence was verified by sequencing (done by Invitrogen, Shanghai, China). We amplified a 688 bp fragment of GFP using primers detailed in Table 1 as control dsRNA (ACY56286; supplied by Zhang Chuan-xi, Institute of Insect Sciences, Zhejiang University).

**Table 1:** PCR primers used in this study.

Primers	Primer sequence	Product size
**For real-time PCR**		
**Q-*NlAC9*-F**	GAACCTGTTGCCTCTCCTCT (59.8°C)	151bp
**Q-*NlAC9*-R**	AAACCAGCAGCACAAGTAGC (57.8°C)	
**Q-*Nlvg*-F**	GTGGCTCGTTCAAGGTTATGG (Tm = 58.0°C)	200bp
**Q-*Nlvg*-R**	GCAATCTCTGGGTGCTGTTG (Tm = 60.2°C)	
**Actin-F**	TGCGTGACATCAAGGAGAAGC (Tm = 60.0°C)	186bp
**Actin-R**	CCATACCCAAGAAGGAAGGCT (Tm = 60.0°C)	
**For dsRNA synthesis**		
***NlAC9*-F**	TAATACGACTCACTATAGGG (T_7_promoter)	579bp
	TAACAGTTTGTCGCAACGCA (Tm = 67.2°C)	
***NlAC9*-R**	TAATACGACTCACTATAGGG (T_7_ promoter)	
	CTGGCGACTGGAATACAAGC (Tm = 69.3°C)	
**For dsRNA synthesis**		
***NlGFP*-F**	TAATACGACTCACTATAGGG (T7promoter)	688bp
	AAGGGCGAGGAGCTGTTCACCG (Tm = 60°C)	
***NlGFP*-R**	TAATACGACTCACTATAGGG (T7protomer)	
	CAGCAGGACCATGTGATCGCGC (Tm = 56°C)	

We used the T_7_ RiboMAXTM Express RNAi System (Promega, Sunnyvale, CA) for dsRNA synthesis, following the Promega instructions. Sense and antisense dsRNAs were produced in separate 20 μL reaction volumes, annealed by mixing and incubating at 70°C for 10 min, and then cooling to room temperature over 20 min. Two microliter RNase A solution (4 mg/ml) and 2 μL RNase-free DNase (1 u/μL) were added to the reaction tube and incubated in a 37°C water bath for 30 min. The dsRNA was precipitated by adding 110 μL 95% ethanol and 4.4 μL 3 M sodium acetate (pH 5.2), then rinsed with 0.5 mL 70% ethanol, dried at room temperature and dissolved in 50 μL nuclease-free water. The purified dsRNAs were quantified by spectroscopy.

dsRNAs were orally delivered to nymphs via an artificial diet [18], with minor modifications to the rearing protocol. RNAi via dsRNA feeding is an effective protocol, inducing a rapid reduction in mRNAs encoding selected BPH genes [15]. In glass-cylinder chambers (15.0- cm high × 2.5-cm diameter), BPHs were fed with three concentrations of dsRNA (0.125, 0.0625, and 0.05) to select the optimal treatment concentration 0.0625μg/μL for this study based on dsRNA-treated silencing efficiency and nymphs mortality (S3 Table). The experimental diets (20 μL) were placed between two layers of stretched Parafilm M membrane enclosed at the two open ends of the chamber, which was replaced every second day. The cylinders were covered with light-tight black cloth, and the two ends loaded with artificial diet were exposed to light. Insects fed on the diets by piercing-sucking the inner Parafilm M membrane of the diet capsule. Experimental insects were transferred into chambers and reared on artificial diets for 24 h before a series of assays. Twenty third instar individuals collected from the trial field were placed in each chamber, and three chambers made up three independent biological replicates. The feeding experiments were carried out in a humidified climate cabinet (90% RH) at 26±2°C with a 16L: 8D photoperiod. Mortality was recorded every other day.

### Effects of dietary dsRNA on biological performance parameters

Our pre-experiments showed that the dsRNA treatments induced 95% mortality in second and third instar nymphs, therefore the third instar nymphs were used for all experiments [18]. When the nymphs reached fifth (final) instar (approximately 8 days), they were collected, released into a glass jar (12-cm high × 6-cm diameter) and maintained with rice plants at the tillering stage under standard conditions until emergence. We paired individual newly-emerged females with newly-emerged males, in three combinations: dsNlAC-treated female × untreated male (♀dsNlAC×♂control), dsGFP-treated female × untreated male (♀dsGFP × ♂control), and untreated female × untreated male (♀control × ♂control). Each pair (♂×♀) was introduced into a glass jar (diameter 6-cm, height 12-cm) with rice stems under standard conditions for oviposition. Fifteen mated females per replicate were maintained to record pre-oviposition period, oviposition period, adult longevity and fecundity. Rice stems were replaced daily during pre-oviposition period, every 2 days during the oviposition period, and every 3 days for female longevity until females died. Eggs inside each rice stem were scraped from the leaf sheaths and blades using a pin to count the number of eggs laid under a microscope. Fecundity of 15 mated pairs was recorded as the average number of total eggs laid. A total of 120 mated females (each treatment) at 2 days post emergence (2 d PE) were collected for determination of fresh body weight, soluble ovary protein content, soluble fat body protein content and ecdysone and JH titers. A part of 15 mated females were collected separately at 2, 3, 5 and 7 d PE to determine induced-*NlAC9* transcript level and dsNlAC9-treated females silencing efficiency posting mating. Another 15 virgin females (each treatment) were recruited separately at 1, 3, 5 and 7 d PE to determine *NlAC9* transcript accumulation and dsNlAC9-treated females silencing efficiency.

### Protein extraction and determinations

Protein was extracted from fat bodies and ovaries as described [19] with slight modification. Ovaries and fat bodies were isolated from ten adults under a zoom stereomicroscope (model XTL20, Beijing Tech Instrument Co., Ltd., Beijing, China) in a cooled petri dish. Ovaries and fat bodies were separately placed in pre-weighed, ice-cold centrifuge tubes and then weighed using a Mettler-Toledo electronic balance (EC100 model; 1/10,000 g sensitivity). A proportional volume of NaCl solution (0.4 M NaCl:1 M phenylmethylsulfonyl fluoride (PMSF), v:w at 20 mL NaCl:1 g ovary or fat body) was added to the tube, the tissues were homogenized on ice, and centrifuged at 16,000×g at 4°C for 20 min. The supernatant was collected after filtering the upper fat layer through glass fibers, mixed with proper amounts of ddH_2_O (1 supernatant: 10 ddH_2_O, v/v), placed at 4°C overnight and centrifuged again at 4,000× g at 4°C for 20 min. The protein sediment was dissolved in 1.5 ml pre-chilled 0.4 M NaCl solution after removing the supernatant. The Bradford method was used to measure protein content using Coomassie Brilliant Blue R250 (Shanghai Chemical Agent Co., Ltd., Shanghai, China) [20]. The absorbance at 595 nm was determined in a UV755 B spectrometer (Shanghai Precision Instrument Co., Ltd., Shanghai, China). Protein quantities were estimated from a BSA standard curve, n = 3 biologically independent replicates (Shanghai Biochemistry Research Institute, Shanghai, China).

### Numbers of yeast-like symbionts (YLS) in fat bodies

Numbers of YLS in fat bodies were measured with a hemacytometer (0.01 mm, 1/400 mm^2^) (25×16 model, Shanghai Qiujing Biochemical Reagent Co., Shanghai, China following Ying and Hou [21]. A total of 45 adult females (15 for each treatment) were collected separately at 2 d post mating. Fat body was dissected from five females in experimental (dsNlAC9 and dsGFP) and control groups, and homogenized in 200 μL saline solution (0.9% NaCl) to generate a mixture from which YSL in 2 μL were counted. The numbers of YLS were counted from 80 mm^2^ squares under a microscope. Each treatment and control was replicated three times.

### Body weights and body size comparison

Replicates for each treatment had 15 females at 2 d PE frozen in liquid nitrogen. The insects were placed in pre-weighed centrifuge tubes and then weighed using a Mettler-Toledo electronic balance, n = 3. Body size from at least fifteen females for each group was photographed with a Leica DMR connected to a Fuji FinePix S2 Pro digital camera (Germany). Scale bar, 200μm.

### JH and ecdysone titers

We followed the instructions from an insect double sandwich ELISA kit for JH and ecdysone determinations (Qiaodu biological technology Co., LTD, Shanghai, China). Each treatment and control was replicated three times (n = 3).

### Ovary dissection

The ovaries were dissected from the 2, 4 and 6 d PE mated females (dsNlAC9-treated × untreated male, dsGFP-treated female × untreated male, and untreated female × untreated male) in 1× phosphate buffered saline (PBS; 137 mM NaCl; 2.68 mM KCl; 1.47 mM KH_2_PO4; and 8.10 mM Na_2_HPO4, pH 7.0), followed by fixation in 3.8% formaldehyde in 1× PBS for 20 min at room temperature. Dissected ovaries were washed with 0.2% Triton-X 100 (Sigma, USA) in 1× PBS three times for 10 min. After washing, ovaries were photographed with a Leica DMR connected to a Fuji FinePix S2 Pro digital camera (Germany). Likewise, after mating with untreated males 2, 4 and 6 d PE, experimental and control females were arrayed and photographed under a microscope. Ovaries from at least ten females for each group were dissected and observed under a microscope. Scale bar, 200μm.

### qRT-PCR analysis

We extracted total RNA from five adult females at 1, 3, 5 and 7 d PE using a SV Total Isolation System Kit (model Z3100, Promega Corporation, Madison, WI, USA). First-strand cDNA was synthesized in a 10 μL reaction volume composed of 0.5 μg RNA, 0.5 μL PrimeScript RT enzyme mix I, 0.5 μL Oligo dT primer (50 μM), 2 μL random hexamers (100 μM), 2 μL 5×PrimeScript Buffer and RNase-free dH2O up to a final volume of 10 μL, following instructions of the PrimeScript RT Kit (TaKaRa Biotechnology, Dalian, China). The cDNA was reverse transcribed at 37°C for 15 min, 85°C for 5 s and 4°C for 5 min. Likewise, total RNA from all experimental (dsNlAC9 and dsGFP) and control females were extracted and reverse transcribed. Two microliters of the first-strand cDNA were amplified by qPCR in 20 μL reaction mixtures using a CFX96 real-time PCR system (Bio-Rad Co. Ltd., California, USA). We designed two qPCR programs. For *NlAC9*, 94°C for 2 min, followed by 40 cycles of 94°C for 5 s, 59.7°C for 30 s and 72°C for 30 s. For *vitellogeningene* (*Nlvg*) (AB353856), 94°C for 2 min, followed by 35 cycles of 94°C for 5 s, 60.4°C for 30 s and 72°C for 30 s. *NlAC9* (NLU001904) and *Nlvg* mRNAs were separately quantified in relation to the stable expression [16] of constitutive actin-1 (EU179846). After amplification, a melting curve analysis was performed in triplicate and the results were averaged. The values were calculated using three independent biological samples and the relative *NlAC9* transcript level was analyzed by 2^-ΔΔCT^ method [22]. Each treatment was replicated three times (five females per replicate).

### Statistical analysis

Before performing an analysis of variance (ANOVA), normality and homogeneity of variance were verified based on a Bartlett test. A one-way ANOVA was performed to analyze biological parameter data. Data presented in figures were expressed as mean ± S.E and the differences between three groups were analyzed using a *t-*test (significance level p < 0.05). All Statistical analysis was conducted manipulating DPS data processing system developed by Tang and Feng [23].

## Results

### *NlAC9* mRNA expression

**Fig 1:**
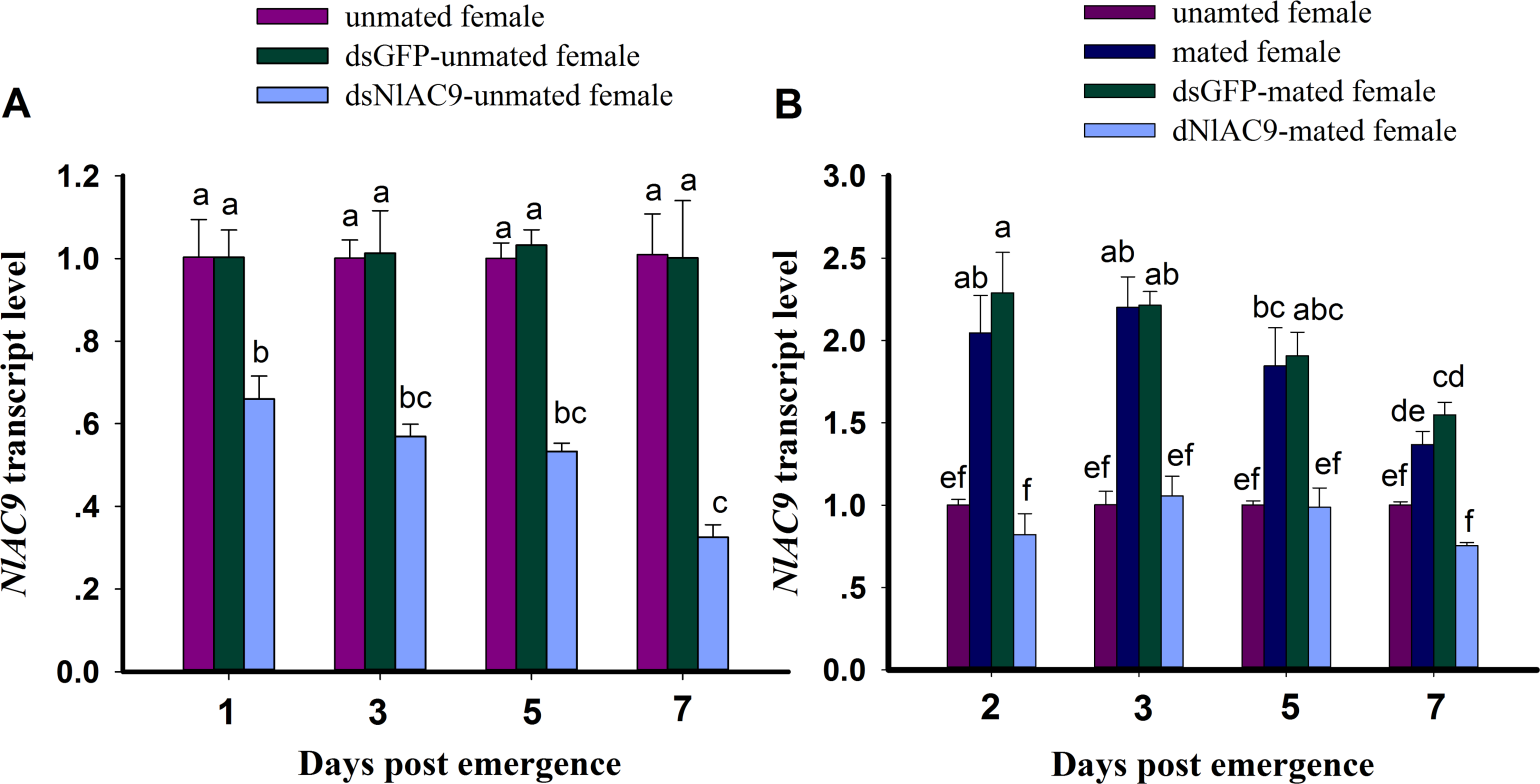
**Relative expression level of *NlAC9* among three treatments at 1, 3, 5, and 7 d PE (A) and among four treatments at 2, 3, 5, and 7 d PE (B).** *NlAC9* expression value of untreated females was converted to 1. The histogram bars denoted mean relative gene expression (n = three independent biological replicates) and data with the same superscript letters were not significantly different at P>0.05. Gene expression was normalized to the β-actin reference gene. PE is post emergence.

Dietary dsNlAC9-unmated femalessignificantly down-regulated transcripts encoding NlAC9 (*F* = 93.1, df = 2, 35, *P* = 0.0001) (Fig 1A). The dietary dsNlAC9-unmated females treatment strongly suppressed mRNA accumulations in adult females, down 34% to 68% compared to untreated controls at 1, 3, 5 and 7 d PE. dsGFP-unmated femalestreatments led to mRNA accumulations similar to controls. There were no significant difference among days post emergence (F = 1.97, df = 3, 35, P = 0.1459). No significant interaction effect between d PE and dsNlAC9-unmated femalestreatments was recorded (*F* = 2.0, df = 6, 35, *P* = 0.1033). *NlPKA* treanscript level (*NlAC9* downstream gene) was significantly also decreased compared to untreated control and dsGFP control group after dsNlAC9-treated at 1d PE (*F* = 40.1, df = 2, 8, *P* = 0.0003) and 3d PE (*F* = 11.2, df = 2, 8, *P* = 0.0095) (S1 Fig).

Dietary dsNlAC9 significantly also suppressed transcript level encoding NlAC9 for mated females (*F* = 211.1, df = 3, 47, *P* = 0.0001) (Fig 1B). The dietary dsNlAC9-mated females strongly suppressed mRNA accumulations in adult females, down from 44.9% to 59.8% compared to mated females at 2, 3, 5 and 7 d PE. dsGFP-treated females post-mating had mRNA accumulations similar to mated females. *NlAC9* gene transcript level were significantly different among days post emergence (d PE) (*F* = 25.4, df = 3, 47, *P* = 0.0001). There was significant difference interaction effect between d PE and dsNlAC9-mated females treatments (*F* = 6.0, df = 9, 47, *P* = 0.0001) (Fig 1B).

### Influence of dietary dsNlAC9 on females

**Fig 2:**
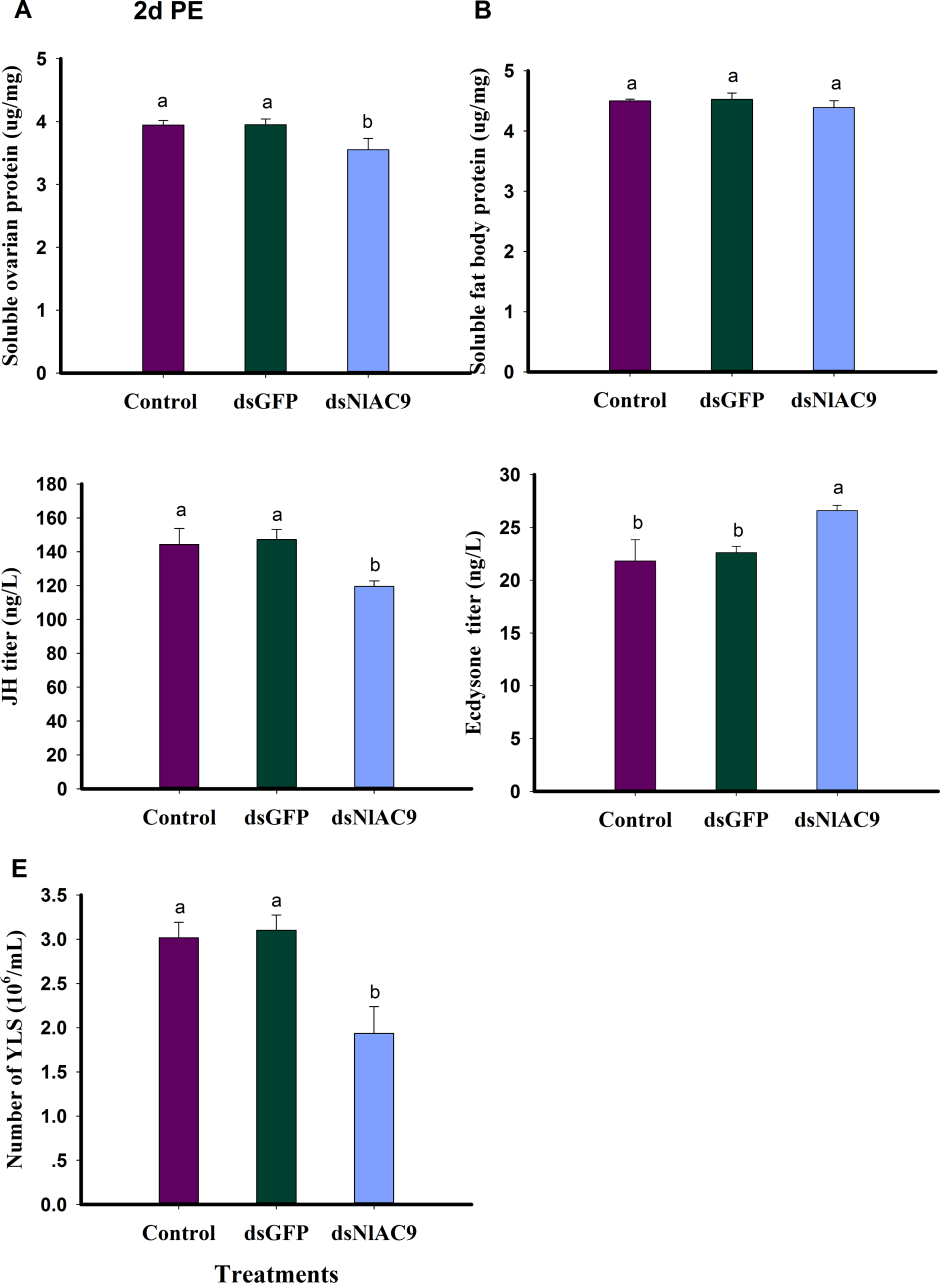
Influence of dietary dsNlAC9 on physiological parameters. (A): soluble ovarian protein content (μg/mg); (B) soluble fat body protein content (μg/mg); (C)JH titer (ng/L); ((D) ecdysone titer (ng/L); (E) number of YLS(10^6^/mL). Every treatment and control was performed in three replicates. Histogram bars represented the mean ± SEM. The bars annotated with the same letter illustrated no significant difference at P>0.05. PE is post-emergence.

Dietary dsNlAC9 treatments led to decreased soluble ovarian protein content (*F* = 11.3, df = 2, 8, *P* = 0.0092), down by 10% (from 3.94 μg/mg to 3.56 μg/mg) compared to untreated and dsGFP treated controls (Fig 2A). The treatments did not influence soluble fat body protein content (*F* = 1.8, df = 2, 8, *P* = 0.2378) (Fig 2B).

We also recorded fluctuations of hormone titer in adult females at 2d PE (Fig 2C and 2D). Dietary dsNlAC increased the ecdysone titer (up 22%) (Fig 2D), and reduced the JH titer (down 17%) (Fig 2C) compared to untreated and dsGFP controls (*F* = 15.2, df = 2, 8, *P* = 0.0045 for JH; *F* = 12.4, df = 2, 8, *P* = 0.0074 for ecdysone). dsNlAC treatment led to significantly decreased YLS abundance at 2 d PE (*F* = 24.7, df = 2, 8, *P* = 0.0013), down by 36% (from 3.02 × 10^6^/mL to 1.93 × 10^6^/mL) compared to controls and by approximately 38% (from 3.10 × 10^6^/mL to 1.93 × 10^6^/mL) relative to dsGFP-treatment (Fig 2E).

### Influence of dietary dsNlAC9 on reproductive parameters

**Fig 3:**
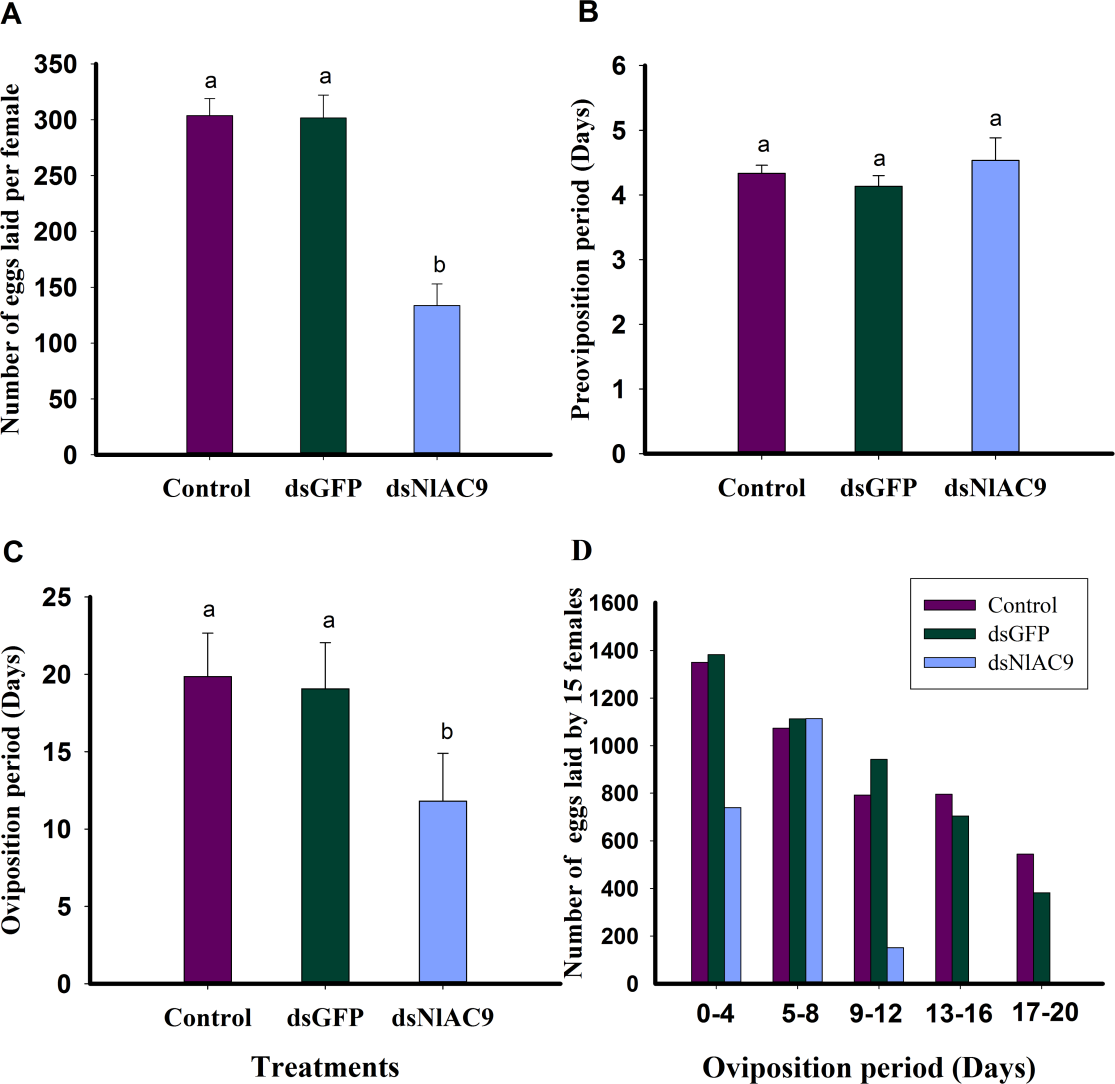
Influence of dietary dsNlAC9 on reproductive parameters. (A) number of eggs laid per female; (B) preovipositon period; (C) ovipostion period. (D) number of eggs laying 15 females. Results were mean ± SEM of three different treatments performed in fifteen replicate. The bars annotated with the same letters reflected no significant difference at P>0.05.

The dietary dsNlAC9 led to significantly decreased number of eggs-laying (*F* = 417.2, df = 2, 44, *P* = 0.0001), down by 56% compared to untreated females (from 303.7 eggs/female to 133.7 eggs/female) and by 55% compared to dsGFP females (from 301.5 eggs/female to 133.7 eggs/female) (Fig 3A).

The pre-oviposition period is the time, in days from newly adult female emergence to the onset of egg-laying, was not prolonged (*F* = 0.7, df = 2, 44, *P* = 0.4907) (Fig 3B). The oviposition period was substantially reduced by 40% (*F* = 33.7, df = 2, 44, *P* = 0.0001), compared to untreated control, and by 38% compared to dsGFP control (from 19.1 days to 11.8 days) (Fig 3C).

The effects of three treatments on numbers of eggs laid by 15 females during oviposition period were presented. We found that numbers of eggs laid by dsNlAC9-treated 15 females (approximately 739 eggs/15 females) was significantly lower compared to untreated control (approximately 1350 eggs/15 females) and dsGFP-treated control (about 1382 eggs/15 females) at 0~4 days in oviposition period (namely 4~8 days posting emergence) (Fig 3D). However, numbers of eggs-laying of dsNlAC9-treated 15 females (about 1114 eggs/15 females) was no significant difference compared to untreated control (approximately 1073 eggs/15 females) and dsGFP-treated control (approximately 1112 eggs/15 females) at 5~8 days in oviposition period (namely 9~12 days posting emergence) (Fig 3D). This result showed that numbers of eggs-laying of dsNlAC9-treated females peaked at 5–8 days in oviposition period. Meanwhile, numbers of eggs-laying of dsNlAC9-treated 15 females (about 151 eggs/15 females) was significantly lower compared to untreated control (approximately 792 eggs/15 females) and dsGFP-treated control (approximately 942 eggs/15 females) at 9~12 days in oviposition period (namely 13~16 days posting emergence) (Fig 3D). Thereafter, dsNlAC9–treated females laid no eggs at 13~16 days in oviposition period (namely 17~20 days posting emergence), and no significance difference between untreated control and dsGFP-treated control was found (Fig 3D).

### dsNlAC9 led to reduced body weight, body size, and longevity

**Fig 4:**
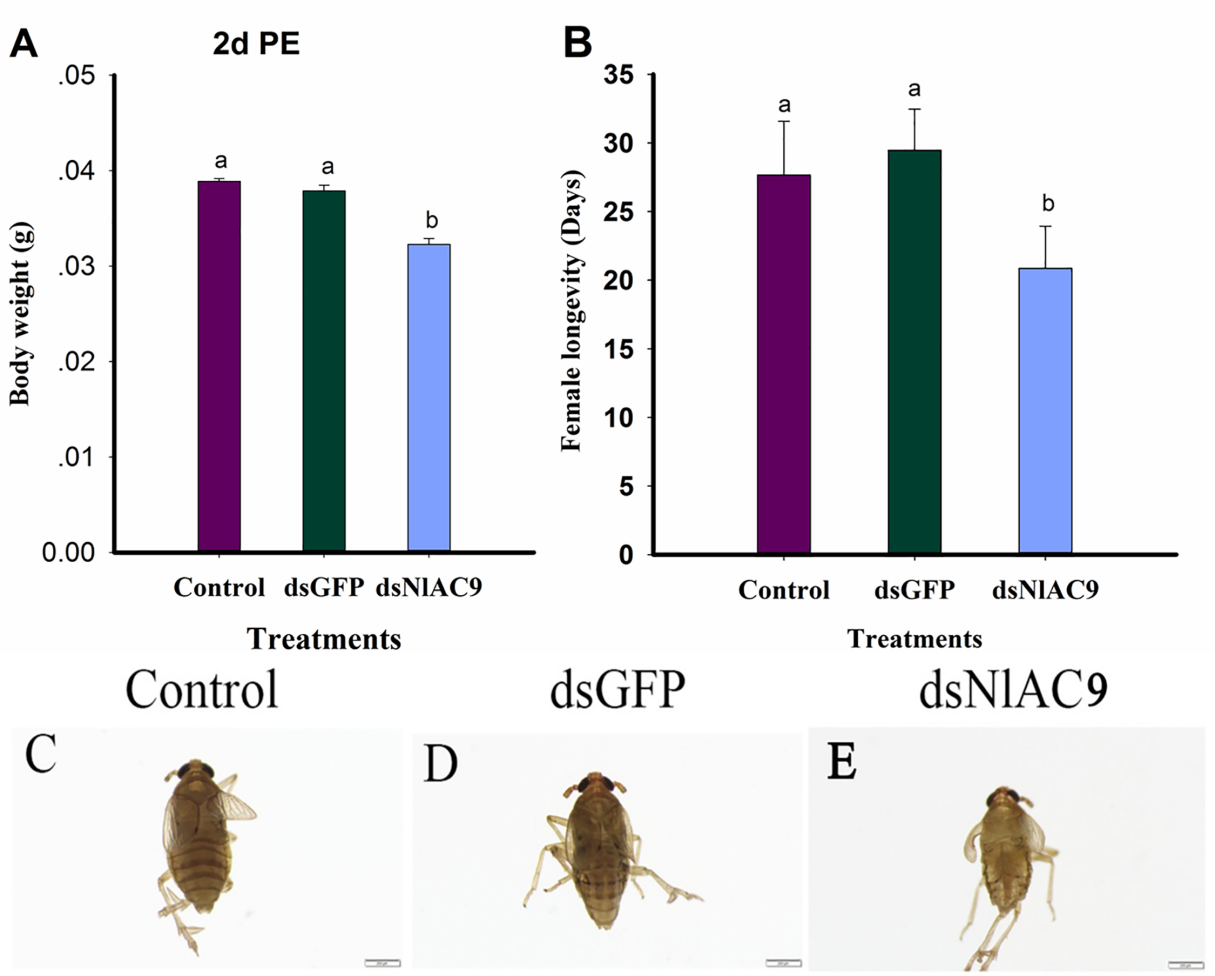
Influence of dietary dsNlAC on body weight, body size, and longevity at 2d PE. (A) body weight (five females/group)(n = three independence replicates); (B) female longevity (n = fifteen independence replicates); (C) body size of control females; (D) body size treated by dsGFP; (E) body size subject to dsNlAC. Histogram bars displayed data in mean ± SEM form. Body size photographed with a Leica DMR connected to a Fuji FinePix S2 Pro digital camera (Germanny). The annotated bars with the same letters presented no significant difference at P>0.05. PE is post-emergence. Body size from at least ten females for each group was compared and photographed under a microscope. Scale bar, 200μm.

The dietary dsNlAC9 treatment led to decreased female body weights (*F* = 48.1, df = 2, 8, *P* = 0.0002), down by 17% compared to untreated control and by 15% compared to dsGFP-treated control at 2 d PE (Fig 4A). Female longevity was reduced by about 25% (*F* = 27.5, df = 2, 44, *P* = 0.0001) compared to untreated control (from 27.7 days to 20.9 days) (Fig 4B). The dietary dsNlAC9 treatment (Fig 4E) led to reduced body sizes compared to untreated control (Fig 4C) and dsGFP treatment (Fig 4D).

### Effect of dietary dsNlAC9 on reproductive systems

**Fig 5:**
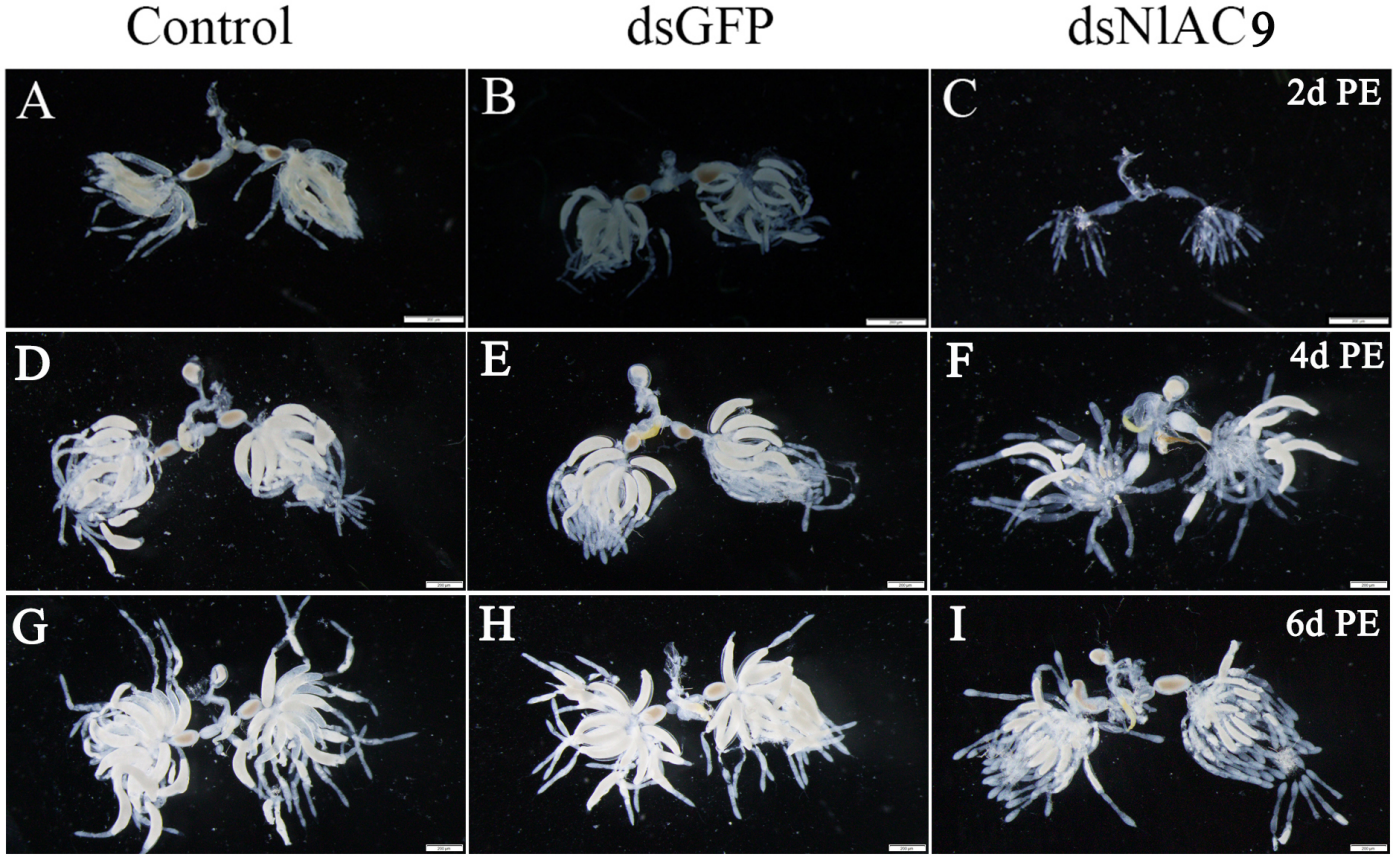
Influence of dietary dsNlAC9 on female reproductive systems at 2, 4 and 6 d PE. The third instar nymphs were treated with dietary dsNlAC9. (A-I) reproductive tracts were dissected from mated females and photographed with a Leica DMR connected to a Fuji FinePix S2 Pro digital camera (Germany). Ovaries from at least ten females for each group were dissected and observed under a microscope. Scale bar, 200μm.

In relation to untreated (Fig 5A, 5D and 5G) and dsGFP-treated controls (Fig 5B, 5E and 5H), dietary dsNlAC9 (Fig 5C, 5F and 5I) inhibited ovary development at 2 d PE, 4 d PE, and 6 d PE. The ovarioles within ovaries of dsGFP-treated and control females contained one or two ripe banana-shaped oocytes. The dietary dsNlAC9 treatments severely inhibited oocyte growth. No mature oocytes were observed at 2 d PE. We observed underdeveloped ovaries with fewer ovarioles and fewer developed eggs in the adults treated with dsNlAC9 (Fig 5F and 5I) at 4 and 6 d PE. In contrast, the control groups showed replete ovarioles and plentiful eggs (Fig 5D and 5G, 5E and 5H) at 4 and 6 d PE.

### Dietary dsNlAC9 led to reduced expression of *Nlvg* mRNA

**Fig 6:**
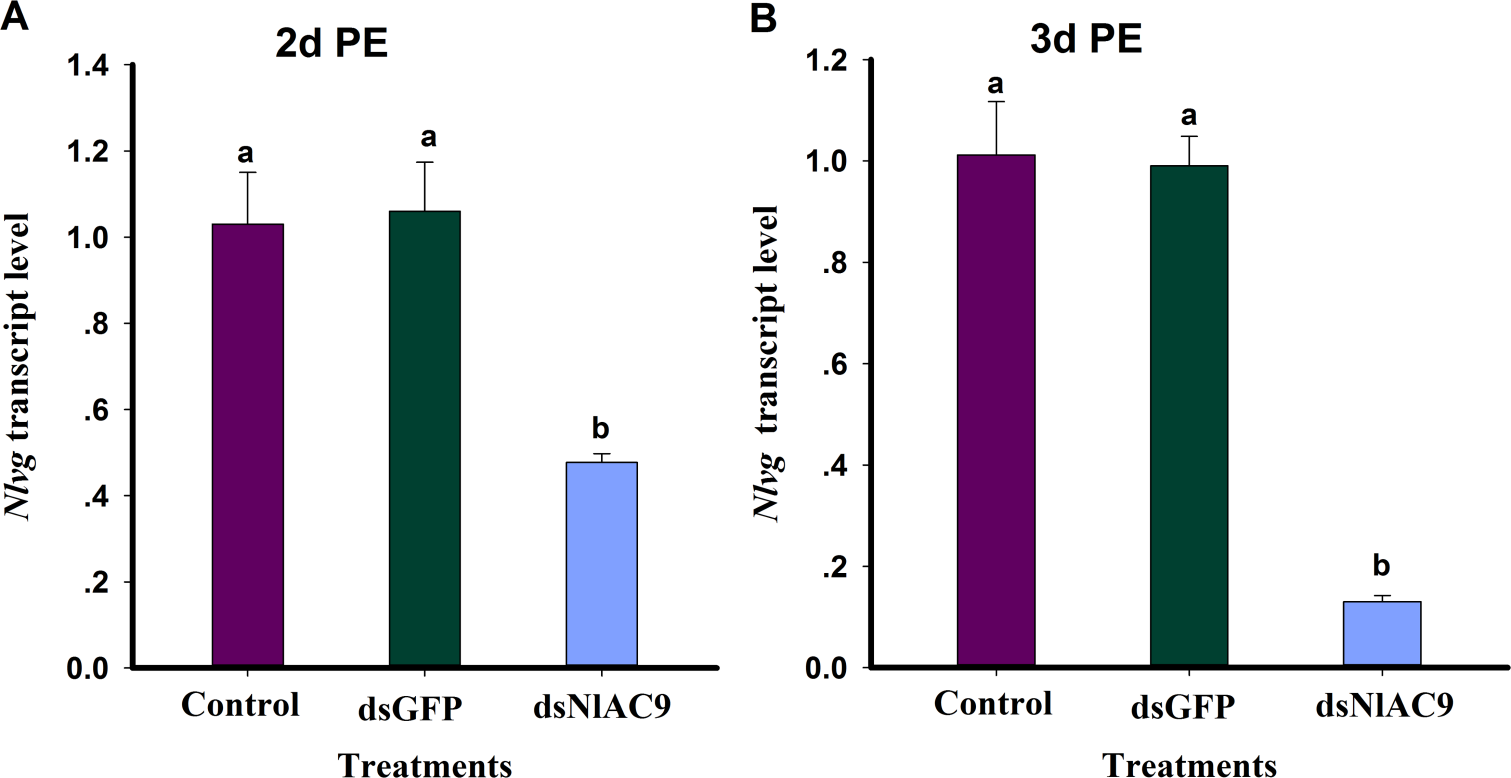
**Influence of dietary dsNlAC9 on transcript level of *Nlvg* at 2d PE (A) and 3d PE (B).** Each treatment and control was repeated three times. The histogram bars show mean relative gene expression (n = three independent biological replicates) and the annotated bars with same letter are not significantly different at P>0.05. Raw gene expression was converted to normalized β-actin reference gene. PE is post-emergence.

Dietary dsNlAC9 substantially reduced *Nlvg* expression in 2 d and 3 d PE (F = 11.5, df = 2, 8, P = 0.0088) (Fig 6A) and 3 DPE (Fig 6B) females (F = 51.7, df = 2,8, P = 0.0002).

## Discussion

The data set forth in this paper strongly support our hypothesis that *Nl*AC9 is a target gene that would lead to reduced BPH fitness and populations. Several points apply. First, dietary dsNlAC9 led to severely decreased mRNAs encoding NlAC9. Second, the experimental treatments led to decreased ovarian protein contents, sizes and development. Third, dietary dsNlAC9 led to meaningful changes in circulating titers of development hormones, ecdysone (increased) and JH (decreased). Fourth, the treatments led to seriously reduced reproductive potentials, recorded as reduced egg laying, oviposition periods and mRNAs encoding the BPH vitellogenin. Fifth, experimental treatments led to reduced female body weights, sizes and longevity. Taken together, the point amount to a very strong argument that targeting *NlAC9* would exert extremely negative impacts on BPH reproduction and populations.

The reduced accumulations of mRNAs encoding NlAC9 would influence a wide range of physiological functions, possibly all functions that are signaled via the AC/cAMP/PKA pathway. Many of these functions lie beyond the scope of this paper, including various homeostatic functions such as water and salt balance, feeding behaviors, digestion, excretion, learning and memory. The AC/cAMP/PKA pathway also acts in insect pathologies. The Cry1Ab toxin, for example, kills insects by binding to a receptor, which activates a cAMP/PKA that leads to a toxin-induced cell death pathway [24]. The reduced reproductive potentials recorded here may be the outcome of direct effects on the parameters we recorded and the indirect effects of overall impaired organismal functioning. Down-regulating *NlAC9* led to increased ecdysone concentration. 20E acts via nuclear receptors, which enhance gene transcription via cAMP-dependent activation of PKA [25]. More to the point, cAMP content in fat body is controlled by ecdysone [26].

dsNlAC9 treatments lead to reduced JH concentrations, which help understand the reduced insect sizes reported here because JH is one of the regulators of adult sizes. Experimentally, reducing JH concentrations by ablating the corpora allata led to undersized larvae [27–30]. The influence of *NlAC9* silencing on decreased JH titers may be seen in reduced Vg synthesis and reduced body weight. Similarly, numbers of eggs laid is an index of fecundity. In BPH, JH stimulates fat body Vg biosynthesis and uptake by the developing oocytes [29]. The reduced JH reported here may directly lead to decreased Vg expression and ovarian uptake. This helps understand how suppressing *NlAC9* leads to smaller body sizes and weights, reduced ovarian development and reproduction at the organismal and population levels.

One indirect effect of suppressing *NlAC9* is the reduced fat body YLS abundances. YLS act in BPH nutrition [31] and loss of the YLS may interfere with reproduction via malnutrition, particularly with respect to nitrogen metabolism. We infer that the increased 20E signaling can exert negative influences at the organismal level. The idea of induced malnutrition may also explain the decreased body weights and sizes we recorded. The malnutrition may develop from reduced populations of YLS and from reduced feeding behavior. Quantitative nutrition is a problematic field and nearly impossible to analyze in very small insects such as BPH. Nonetheless, one of the broader outcomes of suppressing AC/PKA signaling would be seen in behavioral defects, such as feeding. cAMP/PKA signaling acts in feeding in honeybees and other insects [32,33]. We infer that altered feeding behaviors may account, in part, for the reduced body sizes and weights.

In BPH, reduced egg-laying is usually attended by decreased oviposition periods and elongated pre-oviposition periods. Here, dsNlAC9 treatments led to decreased egg-laying and decreased oviposition periods, without altering the pre-oviposition periods. This is an interesting outcome because the reduced AC/PKA signaling led to changes in one reproductive behavior but not the other. The separate effects indicate to us that some, certainly not all, behavioral aspects of BPH reproduction are influenced by AC/PKA signaling.

## Supporting information

S1 Table Analysis of digital gene expression profiles level between mated females and unmated females at 2 d PE. (XLSX)

S2 Table *NlAC9* sequence. (XLSX)

S3 Table Mortality analysis of nmphys feeding on different concentration dsNlAC9+ artificial diet. (XLS)

S1 Fig Effectof dsNlAC9-treated on *NlPKA* gene transcript level at 1 and 3 d PE. (XLSX)

S1 File dsNlAC9 sequence results. (RAR)
